# The effect of inhibition of leukotriene synthesis on the activity of interleukin-8 and granulocyte-macrophage colony-stimulating factor

**DOI:** 10.1155/S0962935193000286

**Published:** 1993

**Authors:** P. J. Roberts, A. R. Pizzey, D. C. Linch

**Affiliations:** Department of Haematology University College London Medical School 98 Chenies Mews London WC1E 6HX UK

## Abstract

The cytokines interleukin-8 (IL-8) and granulocyte-macrophage colony-stimulating factor (GM-CSF) enhanced the extracellular release of arachidonate metabolites from ionophore-stimulated neutrophils by 145 ± 10% (mean ± S.E.M., n = 13) and 182 ± 11% (n = 16), respectively. To determine whether enhanced leukotriene production mediates the effects of these cytokines on neutrophil activity, two different specific arachidonate 5-lipoxygenase (5-LO) inhibitors, piriprost and MK-886, were used to inhibit leukotriene synthesis. Neither inhibitor affected the upregulation of CD11b β_2_-integrin expression or priming of superoxide generation stimulated by IL-8 and GM-CSF. It is concluded that leukotrienes do not mediate either the direct or priming effects of these cytokines and that these classes of anti-inflammatory drugs are therefore unlikely to inhibit the effects of IL-8 and GM-CSF on neutrophil activation.


Mediators of Inflammation 2, 211–216 (1993)

### The effect of inhibition of leukotriene synthesis on the activity of interleukin-8 and granulocyte-macrophage colony-stimulating factor

**P. J. Roberts,[CA] A. R. Pizzey and D. C. Linch**

Department of Haematology,
University College London Medical School,
98 Chenies Mews, London WC1E 6HX, UK

[CA] Corresponding Author

**THE cytokines interleukin-8 (IL-8) and granulocyte-macrophage colony-stimulating factor (GM-CSF) enhanced the extracellular release of arachidonate metabolites from ionophore-stimulated neutrophils by 145 ± 10% (mean ± S.E.M., $n = 13$) and 182 ± 11% ($n = 16$), respectively. To determine whether enhanced leukotriene production mediates the effects of these cytokines on neutrophil activity, two different specific arachidonate 5-lipoxygenase (5-LO) inhibitors, piriprost and MK-886, were used to inhibit leukotriene synthesis. Neither inhibitor affected the upregulation of CD11b $\beta_2$-integrin expression or priming of superoxide generation stimulated by IL-8 and GM-CSF. It is concluded that leukotrienes do not mediate either the direct or priming effects of these cytokines and that these classes of anti-inflammatory drugs are therefore unlikely to inhibit the effects of IL-8 and GM-CSF on neutrophil activation.**

**Key words:** Granulocyte-macrophage colony-stimulating factor, $\beta_2$-Integrin, Interleukin-8, Leukotrienes, 5-Lipoxygenase inhibitors, Neutrophil, Superoxide

### Introduction

The cytokines granulocyte-macrophage colony-stimulating factor (GM-CSF) and interleukin-8 (IL-8) are potent modulators of neutrophil function. Both GM-CSF and IL-8 stimulate increased $\beta_2$-integrin expression,[1–3] a direct effect. They also have indirect effects, for example, at concentrations that are not directly stimulatory, they enhance or 'prime' superoxide production subsequently stimulated by a second agonist.[1,4–6]

The biochemical pathways which these cytokines use are not fully elucidated. Phospholipase $A_2$ activity may mediate some effects of GM-CSF, as this cytokine stimulates both the release of metabolites from phospholipids labelled with $^3$H-arachidonate[7–9] and the synthesis of platelet-activating factor.[10–12] However, IL-8 does not trigger the synthesis of these compounds.[13–15] 5-Lipoxygenase (5-LO) activity may also mediate GM-CSF effects as GM-CSF directly stimulates the release of leukotriene $B_4$ ($LTB_4$),[10] although others have not confirmed these observations.[12,16] Exogenous $LTB_4$ increases phagocyte CD11b $\beta_2$-integrin expression,[17] therefore increased eicosanoid synthesis may mediate the GM-CSF directed upregulation of CD11b. IL-8 does not directly stimulate $LTB_4$ synthesis, but can do so when cells are given exogenous arachidonate 5 min before stimulation with IL-8.[13]

However, both IL-8 and GM-CSF increase 5-LO activity in response to other agonists,[8,12,16,18] and this may contribute to the priming effect of these cytokines on the respiratory burst.[6] This is because the products of 5-LO, 5-hydroxyeicosatetraenoic acid (5-HETE)[19] and $LTB_4$[20,21] themselves potentiate superoxide production. A recent study of respiratory burst priming showed that partial inhibition of 5-LO, did not prevent respiratory burst priming by either TNF$\alpha$ or GM-CSF,[12] but because $LTB_4$ release was inhibited by less than 70%, these data are inconclusive.

To examine whether leukotriene synthesis is involved in either the direct or indirect priming activities of GM-CSF and IL-8, measurements were made of cytokine-stimulated changes in the expression of the $\beta_2$-integrin CD11b, and cytokine priming of superoxide generation in human neutrophils pretreated with and without either the arachidonate 5-LO inhibitor, 6,9-deepoxy-6,9-(phenylimino)-delta$^{6,8}$-prostaglandin $I_1$, (piriprost),[22] or the five-lipoxygenase activating protein (FLAP) inhibitor, 3-[1-(*p*-chlorobenzyl)-5-isopropyl-3-*t*-butylthioindol-2-yl]-2,2′-dimethylpropanoic acid, (MK-886).[23] The authors show that cells whose leukotriene synthesis is virtually abolished have intact responses to both cytokines.

### Materials and Methods

*Materials:* Recombinant human (rh) IL-8 from monocytes was a kind of gift of Sandoz Forschungsinstitut, Vienna, Austria. A stock solution at 100 μg/ml was prepared in sterile PBS with 2% foetal calf serum and stored at −80°C. A

© 1993 Rapid Communications of Oxford Ltd

1 μg/ml stock solution of rhGM-CSF (expressed in *E. coli* and provided by Hoechst UK, Beringwerke, Marburg, Germany) was prepared in RPMI medium with 2% (v/v) foetal calf serum and stored at −20°C. Lymphoprep (density = 1.077 g/ml) was obtained from Nycomed Pharma AS, Oslo, Norway. Dulbecco's phosphate buffered saline (PBS) was from Gibco BRL, Paisley, Scotland. The Mo1 CD11b and control IgM antibodies (Coulter clone) were from Coulter Electronics Ltd., Luton, Bedfordshire, UK. [5,6,8,9,11,12,14,15-$^3$H]arachidonic acid was from Amersham International, Amersham, Buckinghamshire, UK. Piriprost (6,9-deepoxy-6,9-(phenylimino)-delta$^{6,8}$-prostaglandin $I_1$) was a gift from Dr Michael Bach, The Upjohn Laboratories, Kalamazoo, MI, USA. The FLAP antagonist, MK-886 (L-663,536) 3-[1-(*p*-chlorobenzyl)-5-isopropyl-3-*t*-butylthioindol-2-yl]-2,2′-dimethylpropanoic acid, was a gift from Dr Gillard, Merck-Frosst Canada Inc., Pointe Claire-Dorval, Quebec, Canada. C18 (300 mg) Maxi-Clean columns were from Alltech, Carnforth, Lancashire, UK. Leukotriene standards were from Cascade Biochem Ltd, Reading, Berkshire, UK.

*Neutrophil purification:* Neutrophils were purified from venous blood anticoagulated with 2 mM EDTA, pH 7.4 by dextran sedimentation, Lymphoprep density gradient separation and erythrocyte lysis, as described previously.[4] The purified neutrophils were suspended in PBS supplemented with 0.9 mM $CaCl_2$, 0.5 mM $MgCl_2$ and 5 mM D-glucose (PBSG).

*CD11b expression:* 50 μl aliquots of purified neutrophils (1 × $10^6$/ml) were incubated with either diluent, IL-8 (100 ng/ml), or GM-CSF (10 ng/ml) for 30 min at 37°C. The samples were placed on ice and CD11b expression was measured by flow cytometry using direct immunofluorescence with a mouse IgM FITC-conjugated CD11b monoclonal antibody (Mo1) as described previously.[24]

*Superoxide generation:* Cells (1 × $10^6$/ml) were equilibrated in 1 ml plastic spectrophotometer cuvettes for 5 min before addition of cytokines or diluent. Cells were stimulated with either 1 μM formyl-methionyl-leucyl-phenyalanine (fMLP) or 1.67 μM 12-*O*-tetradecanoylphorbol-13-acetate (TPA). Superoxide production by purified neutrophils at 37°C was measured by the continuous assay of superoxide dismutase-inhibitable reduction of ferricytochrome c, as described previously.[4]

*Extracellular release of [$^3$H]arachidonate-labelled metabolites from intracellular arachidonate:* Purified neutrophils (5 × $10^6$/ml PBSG, 0.1% FCS) were labelled with [$^3$H]arachidonate (202 Ci/mmol) at a final concentration of 0.5 μCi/ml, for 2 h at 20°C with occasional mixing. Under these conditions approximately 80% of the isotope was taken into the cells. The radiolabelled cells were centrifuged (180 × *g*, 5 min) and the supernatant containing unincorporated isotope was removed. The cell pellet was washed four times in PBSG and finally resuspended to 2 × $10^6$ cells/ml PBSG. Control experiments measuring superoxide production showed that this labelling procedure did not itself cause priming of neutrophils. 0.5 ml aliquots were equilibrated to 37°C, mixed with either diluent, IL-8 (100 ng/ml), or GM-CSF (10 ng/ml) and incubated for 30 min at 37°C. Arachidonate metabolism was stimulated with $Ca^{2+}$ ionophore A23187 (1 μM) for 7 min and the reaction was terminated by placing the samples on ice. The samples were centrifuged (12 000 × *g*, 2 min) and 0.4 ml aliquots of the supernatants were assayed for radioactivity by liquid scintillation spectroscopy. The cell pellets were lysed with 0.1% Triton-X-100, made up to 0.5 ml volume, and 0.4 ml aliquots were also assayed for radioactivity.

*Inhibitors:* An 8.7 mM stock solution of the 5-LO inhibitor, piriprost,[22] in distilled water and a 100 μM stock solution of the FLAP antagonist, MK886,[23] in DMSO, were prepared immediately prior to use. In all experiments described neutrophils were incubated for 5 min with inhibitors before priming with cytokines or stimulation with 1 μM A23187. Dose–response experiments measuring the release of metabolites labelled with [$^3$H]arachidonate were performed to determine the optimal final concentration of inhibitors. Control experiments showed that the final concentration of DMSO used (0.1% v/v) had no effect on neutrophil function.

*Measurement of leukotriene synthesis:* Neutrophils (1 × $10^7$/ml, 4 × $10^7$/sample) were warmed to 37°C and incubated with MK-886 or diluent as described above and then incubated with or without GM-CSF (10 ng/ml) for 30 min before stimultion with 1 μM A23187. The reaction was stopped after 10 min by addition of 2 vol of ice cold ethanol, followed by 200 ng prostaglandin $B_2$ as internal standard. The samples were cooled on ice. The samples were diluted with water to give a final ethanol concentration of 15% (v/v) and centrifuged at 400 × *g* for 10 min at 4°C. The supernatants were acidified with formic acid to pH 3.0. Solid phase extraction of leukotrienes was performed using C18 minicolumns following the method of Powell.[25] Eicosanoids were eluted with 5 ml ethyl acetate. The samples were dried by centrifugal evaporation, resuspended in 0.5 ml ethyl acetate and transferred to a 1.5 ml polypropylene container. The samples were again dried and the pellet was dissolved in 50 μl methanol–water (70 : 30). Eicosanoids were separated by reverse-phase HPLC (RP-HPLC) using a Spherisorb C18 column (ODS2, 250 ×

4.6 mm i.d., Phillips Scientific, Cambridge, UK) attached to a Waters 625 LC system (Millipore UK Ltd., Watford, Hertfordshire, UK) with a 20 $\mu$l injection loop. The isocratic mobile phase was methanol : water : acetic acid (67 : 33 : 0.1 by vol.), pH 6.25. The flow rate was 0.75 ml/min and the effluent was monitored by a Waters 991 photodiode array detector. Chromatograms were recorded at 270 nm and the eluant peaks were scanned from 190–320 nm. After 60 min, methanol : water (90 : 10) was mixed with the mobile phase in a proportion of 1 : 1, to ensure that all eicosanoids were eluted from the column. Peaks obtained were compared with authentic leukotriene standards.

*Statistical analysis:* Unless otherwise indicated the standard error of the estimate of the mean value is given and the statistical test used is the Student's *t*-test for paired samples.

### Results

*Priming of neutrophil 5-LO activity by IL-8 and GM-CSF:* The major metabolites of endogenous arachidonate in neutrophils stimulated with the $Ca^{2+}$ ionophore, A23187, are the 5-LO products, 5-HETE and $LTB_4$.[22] The synthesis of metabolites from endogenous [$^3$H]arachidonate pools can be estimated by counting the extracellular radioactivity released from stimulated cells. Pilot experiments using thin layer chromatography showed that under the labelling conditions used 63% of incorporated [$^3$H]arachidonate was in glycerophospholipid pools with no detectable free intracellular [$^3$H]arachidonate. Table 1 shows the effects of the cytokines, IL-8 and GM-CSF on the release of radioactivity from both resting and ionophore-stimulated neutrophils. The release of radioactivity stimulated by IL-8 was not different from control, whereas the release stimulated by GM-CSF was greater than control by 200 cpm/10$^6$ cells (representing 0.5% of total incorporated radioactivity), a very minor but statistically significant difference ($p = 0.003$, $n = 13$). In samples preincubated for 30 min with cytokine diluent, 1 $\mu$M A23187 stimulated the release of about 4000 cpm/10$^6$ cells greater than background, which represented on average 9.5% of total incorporated radioactivity. When neutrophils were incubated with IL-8 and GM-CSF before stimulation, the release of radioactivity in response to A23187 was enhanced by 145 $\pm$ 10% ($n = 13$) and 182 $\pm$ 11% ($n = 16$), respectively, a statistically significant increase.

**Table 1.** Effect of IL-8 and GM-CSF on the extracellular release of radioactivity from neutrophil phospholipids with [$^3$H]arachidonate

| Stimulus | Release of [$^3$H]arachidonate metabolites[a] (cpm/10$^6$ cells) | |
|---|---|---|
| | IL-8 (100 ng/ml) | GM-CSF (10 ng/ml) |
| Diluent + 0.01% DMSO | 990 $\pm$ 116[b] ($p = 0.18$) | 929 $\pm$ 94 ($p = 0.003$) |
| Cytokine + 0.01% DMSO | 1 062 $\pm$ 142 | 1 132 $\pm$ 130 |
| Diluent + 1 $\mu$M A23187 | 4 514 $\pm$ 592 ($p = 0.001$) | 5 040 $\pm$ 773 ($p = 0.001$) |
| Cytokine + 1 $\mu$M A23187 | 5 977 $\pm$ 815 | 8 505 $\pm$ 1272 |

[a]Radiolabelled neutrophils were incubated at 37°C for 30 min with either IL-8, GM-CSF or 2% FCS diluent, and then stimulated with either A23187 or DMSO diluent.
[b]The data shown are the mean $\pm$ S.E.M. for 13 experiments with IL-8 and 16 experiments with GM-CSF. The significance values ($p$) of the differences between paired samples treated with and without growth factor are given in parentheses.

*Inhibition of 5-LO activity by piriprost and MK-886:* Experiments were performed to determine the optimal concentrations of the 5-LO inhibitors, piriprost and MK-886, in which the release of [$^3$H]arachidonate-labelled metabolites from ionophore-stimulated cells was determined as above. Neutrophils were incubated for 30 min with the inhibitors at a range of concentrations followed by stimulation with 1 $\mu$M A23187. Figure 1 shows that almost complete inhibition of the release of radioactivity from unprimed neutrophils was achieved by piriprost and MK-886. The mean $IC_{50}$ for piriprost was 6.3 $\pm$ 1.8 $\mu$M ($n = 3$) and for MK-886 was 5.5 $\pm$ 1.7 nM ($n = 4$). In six experiments performed at a single concentration, the mean inhibition of radioactive release from unprimed neutrophils was 93 $\pm$ 2% with 100 nM MK-886 and 95 $\pm$ 1% with 87 $\mu$M piriprost. The release of radioactivity from IL-8 primed cells was similarly inhibited by 95 $\pm$ 1% ($n = 5$) with 100 nM MK-886 and by 95 $\pm$ 2% ($n = 3$) with 87 $\mu$M piriprost. To confirm that eicosanoid synthesis was inhibited in GM-CSF-primed cells further studies were performed.

*Confirmation of the inhibition of 5-lipoxygenase in GM-CSF primed cells by piriprost and MK-886 using RP-HPLC:* The authors have previously shown using RP-HPLC that total cellular leukotriene synthesis and extracellular release by ionophore-stimulated neutrophils is completely inhibited by 87 $\mu$M piriprost.[26] In the present study total eicosanoid production was similarly measured in ionophore-stimulated cells by extracting both the cells and supernatants together as described in the methods and then separating the leukotrienes by RP-HPLC. Neutrophils were incubated with either 100 nM MK-886 or 0.1% DMSO for 5 min before exposure to GM-CSF (10 ng/ml) or diluent for 30 min. All samples were stimulated with 1 $\mu$M A23187 for 10 min. In samples not exposed to MK-886, peaks were identified that had the spectral characteristics of $LTB_4$ and its oxidation products, however the

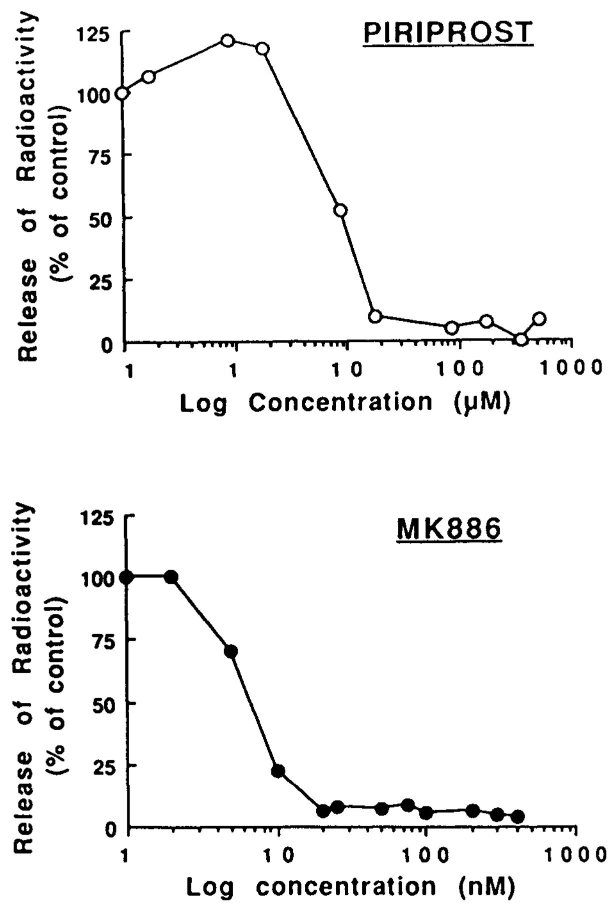

FIG. 1. Dose-dependent inhibition by piriprost and MK-886 of the release of [$^3$H]arachidonate metabolites from neutrophils stimulated with 1 μM A23187. Cells ($2 \times 10^6$/ml) were incubated with inhibitors or their respective diluents ($H_2O$ and 0.2% DMSO), for 5 min at 37°C before stimulation. Background release was subtracted and the data expressed as a percentage of the release from control samples incubated with diluent. The data shown are the mean values of duplicate samples from single experiments representative of three performed with piriprost and four with MK-886.

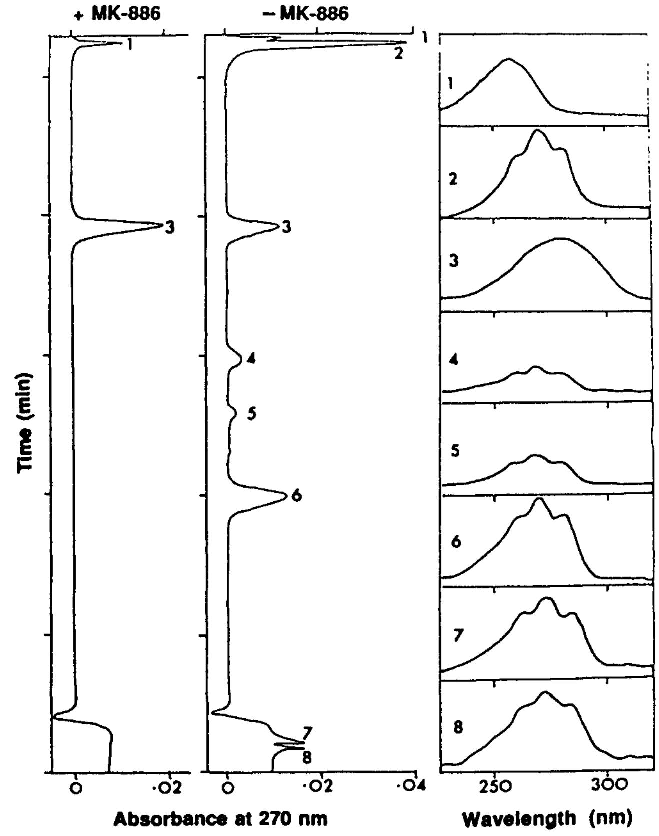

FIG. 2. Inhibition of leukotriene synthesis by MK-886 in GM-CSF primed neutrophils, analysed by RP-HPLC. The two left-hand columns are chromatograms recorded at 270 nm for neutrophils pre-treated with (+MK-886) and without (−MK-886) 100 nM MK886, before priming with GM-CSF and stimulation with 1 μM A23187. Spectral analysis of the eluant peaks scanned from 220 to 320 nm is shown in the right-hand column. The peaks shown are: (1) unidentified peak present in all samples ($UV_{max}$ 258 nm); (2) 20-hydroxy-$LTB_4$ ($UV_{max}$ 270 nm); (3) prostaglandin $B_2$ ($UV_{max}$ 278 nm); (4) and (5) $LTB_4$ metabolites ($UV_{max}$ 270 nm); (6) $LTB_4$; (7) and (8) further $LTB_4$ metabolites eluted from the column with methanol ($UV_{max}$ 270 nm).

peptidoleukotrienes, $LTC_4$, $LTD_4$ and $LTE_4$ were not detected. Figure 2 shows the data for GM-CSF primed neutrophils and similar data (not shown) were obtained with unprimed cells. Figure 2 also shows the complete inhibition of leukotriene production in GM-CSF primed neutrophils pre-incubated with 100 nM MK-886. Similar inhibition of leukotriene synthesis by MK-886 was seen in unprimed neutrophils (data not shown).

*The effect of 5-LO inhibitors on the activity of IL-8 and GM-CSF:*

*a) Priming of fMLP-stimulated superoxide generation:* Neutrophil superoxide production was measured by the superoxide dismutase-inhibitable reduction of ferricytochrome c. Initial experiments showed that the 5-LO inhibitors, piriprost (87 μM) and MK-886 (100 nM), did not inhibit respiratory burst activity stimulated by 1.67 μM TPA, demonstrating that the doses used were not toxic to the cells and that these inhibitors did not inhibit protein kinase c (data not shown). Neither did piriprost nor MK-886 inhibit the generation of superoxide stimulated by 1 μM fMLP (Table 2). Table 2 also shows that in experiments where GM-CSF and IL-8 increased fMLP-stimulated superoxide production by between three to five-fold, there was no reduction in this priming activity when replicate samples were preincubated with either piriprost or MK-886 before exposure to the cytokines, conditions which were demonstrated in parallel experiments to give greater than 90% inhibition of 5-LO activity.

*b) Upregulation of CD11b expression:* Neutrophil CD11b expression was measured by direct immunofluorescence, using flow cytometry to determine the mean cell fluorescence (MCF). Figure 3 shows that IL-8 rapidly stimulated the increased expression of CD11b antigen on purified neutrophils. CD11b expression was upregulated more slowly by GM-CSF, reaching a maximum by 20 min. When cells were pre-incubated with either 100 nM MK-886 or 87 μM piriprost before exposure to cytokines, there was no change in either the kinetics or magnitude of the response (Fig. 3).

**Table 2.** Effect of 5-lipoxygenase inhibitors on the priming of the fMLP-stimulated neutrophil respiratory burst by IL-8 and GM-CSF[a]

| Treatment | Superoxide generation ($nmol/10^6$ cells) | |
|---|---|---|
| | −Cytokine | +Cytokine |
| 0.2% DMSO ± GM-CSF | 5.4 ± 1.1[b] | 27.3 ± 2.7 (506%)[c] |
| MK-886 ± GM-CSF | 4.8 ± 0.7 | 26.1 ± 1.9 (544%) |
| $H_2O$ ± GM-CSF | 5.2 ± 1.2 | 14.3 ± 1.8 (275%) |
| Piriprost ± GM-CSF | 5.0 ± 1.0 | 18.3 ± 3.2 (366%) |
| 0.2% DMSO ± IL-8 | 5.6 ± 2.0 | 20.2 ± 7.4 (361%) |
| MK-886 ± IL-8 | 6.4 ± 0.8 | 19.8 ± 7.1 (309%) |

[a]Cells were incubated with either 87 μM piriprost, 100 nM MK-886 or diluent before the addition of GM-CSF (10 ng/ml) or IL-8 (100 ng/ml), followed by stimulation with 1 μM fMLP.
[b]The absolute data shown are the mean ± 1S.E. of seven experiments with GM-CSF and three experiments with IL-8.
[c]Superoxide production by samples treated with cytokine was expressed as a percentage of control (−cytokine) and is given in parentheses.

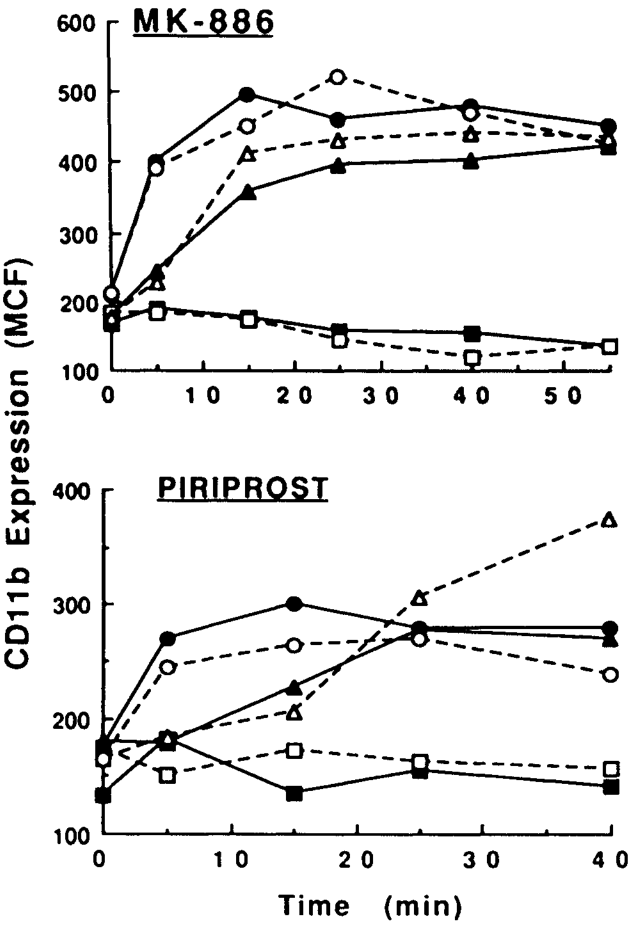

FIG. 3. Effect of 5-LO inhibition on the upregulation of CD11b expression stimulated by IL-8 and GM-CSF. Neutrophils were incubated with the inhibitors (+I) 100 nM MK-886, 87 μM piriprost, or diluent, for 5 min before stimulation with either 100 ng/ml IL-8, 10 ng/ml GM-CSF, or cytokine diluent. The mean cell fluorescence is given in arbitrary units. Values shown are the mean of duplicate samples from single experiments, representative of two that were performed. The range of the duplicate samples was on average 20% of the mean. △, GM-CSF + I; ▲, GM-CSF; ○, IL-8 + I; ●, IL-8; □, control + I; ■, control.

### Discussion

The authors have shown that both IL-8 and GM-CSF enhance or 'prime' the $Ca^{2+}$ ionophore-stimulated extracellular release of [$^3$H]-arachidonate metabolites from endogenous phospholipid pools. This confirms previous studies using GM-CSF.[7–9] Daniels *et al.*[15] have recently shown that IL-8 can prime the release of [$^3$H]arachidonate from cytochalasin B-treated neutrophils, however this fungal metabolite grossly alters neutrophil phospholipid metabolism and is itself a priming agent.[27]

Using specific inhibitors of arachidonate 5-lipoxygenase, piriprost,[22] and the indole derivative, MK-886,[23] which specifically binds to the membrane protein FLAP, inhibiting translocation of 5-LO and leukotriene production in intact cells,[28] it has been demonstrated that the release of [$^3$H]arachidonate metabolites from unprimed or IL-8 primed neutrophils could be blocked by greater than 90%. That these compounds inhibited both the intracellular synthesis and extracellular release of leukotrienes in unprimed and GM-CSF primed cells was confirmed by RP-HPLC. In this study the $Ca^{2+}$ ionophore A23187 was used to demonstrate the efficacy of these compounds because it is the most potent stimulus for neutrophil leukotriene production. The inhibition observed with these compounds was not due to any toxic effect because at the same concentrations neither inhibited the generation of superoxide stimulated by phorbol ester or fMLP.

The authors sought to determine whether leukotrienes mediate the signal transduction or priming activities of GM-CSF and IL-8. Previous studies have shown little evidence for the involvement of 5-LO in IL-8 signalling, but studies of Rapoport *et al.*[6] have suggested a role for this enzyme in GM-CSF signal transduction. Our data exclude the possibility that leukotrienes function in this respect, as the upregulation of cell adhesion molecules directly stimulated by IL-8 and GM-CSF was not altered by complete inhibition of 5-LO. Priming of the fMLP stimulated respiratory burst by IL-8 and GM-CSF was similarly unaffected by inhibition of 5-LO.

It is concluded that leukotriene synthesis does not mediate either the direct activity or the priming effects of IL-8 and GM-CSF on mature neutrophils. The study shows that while therapeutic treatment of inflammation with 5-lipoxygenase inhibitors will inhibit leukotriene production by neutrophils exposed to cytokines, it is unlikely to inhibit other activities of these cytokines such as those associated with upregulation of cell adhesion proteins, degranulation, adhesion and migration, or increased oxygen free-radical production.

### References

1. Lopez AF, Williamson DJ, Gamble JR, *et al.* Recombinant human GM-CSF stimulates *in vitro* mature human neutrophil and eosinophil function, surface receptor expression and survival. *J Clin Invest* 1986; **78:** 1220–1228.
2. Devereux S, Bull HA, Campos-Costa D, Saib R, Linch DC. GM-CSF induces changes in cellular adhesion molecule expression and adhesion to endothelium; *in-vitro* and *in-vivo* studies in man. *Br J Haematol* 1989; **71:** 323–330.
3. Detmers PA, Lo SK, Olsen-Egbert E, Walz A, Baggiolini M, Cohn ZA. NAP 1/IL-8 stimulates binding activity of the leukocyte adhesion receptor CD11b/CD18 on human neutrophils. *J Exp Med* 1990; **171:** 1155–1162.
4. Roberts PJ, Devereaux S, Pilkington GR, Linch DC. FcγRII-mediated superoxide production by phagocytes is augmented by GM-CSF without a change in FcγRII expression. *J Leuk Biol* 1990; **48:** 247–257.
5. Yuo A, Kitagawa S, Kasahara T, Matsushima K, Saito M, Takaku F. Stimulation and priming of human neutrophils by IL-8; cooperation with tumor necrosis factor and colony-stimulating factors. *Blood* 1991; **78:** 2708–2714.
6. Rapoport AP, Abboud CN, DiPersio JF. GM-CSF and G-CSF: Receptor biology, signal transduction and neutrophil activation. *Blood Rev* 1992; **6:** 43–57.
7. Sullivan R, Griffin JD, Simons ER, *et al.* Effects of human granulocyte and macrophage colony-stimulating factors on signal transduction pathways in human granulocytes. *J Immunol* 1987; **139:** 3422–3430.
8. DiPersio JF, Billing P, Williams R, Gasson JC. Human GM-CSF and other cytokines prime human neutrophils for enhanced arachidonate release and leukotriene $B_4$ synthesis. *J Immunol* 1988;m **140:** 4315–4322.
9. Atkinson YH, Murray AW, Krilis, Vadas MA, Lopez AF. Human tumor necrosis factor-alpha directly stimulates arachidonic acid release in human neutrophils. *Immunology* 1990; **70:** 82–87.
10. DiPersio JF, Aggarwal S, Golde DW. GM-CSF directly induces platelet activating factor synthesis: activation of lysoPAF acetyltransferase. *Blood* 1990; **76:** 179a (abstract 707).
11. DeNichilo MO, Stewart AG, Vadas MA, Lopez AF. GM-CSF is a stimulant of PAF and superoxide anion generation by human neutrophils. *J Biol Chem* 1991; **266:** 4896–4902.
12. Stewart AG, Harris T, DeNichilo M, Lopez AF. Involvement of leukotriene $B_4$ and platelet-activating factor in cytokine priming of human polymorphonuclear leucocytes. *Immunology* 1991; **72:** 206–212.
13. Schroder J-M. The monocyte-derived neutrophil activating peptide (NAP/interleukin 8) stimulates human neutrophil arachidonate-5-lipoxygenase, but not the release of cellular arachidonate. *J Exp Med* 1989; **170:** 847–863.
14. Wirthmueller U, Baggiolini M, de Weck AL. Receptor-operated activation of polymorphonuclear leukocytes: different effects of NAP-1/IL-8 and fMet-Leu-Phe or C5a. *Biochem Biophys Res Commun* 1991; **176:** 972–978.
15. Daniels RH, Finnen MJ, Hill ME, Lackie JM. Recombinant human monocyte IL-8 primes NADPH-oxidase and phospholipase $A_2$ activation in human neutrophils. *Immunology* 1992; **75:** 157–163.
16. McColl SR, Krump E, Naccache PH, Borgeat P. Enhancement of human neutrophil leukotriene synthesis by human GM-CSF. *Agents & Actions* 1989; **27:** 465–468.
17. Miller LJ, Bainton DF, Borregaard N, Springer TA. Stimulated mobilization of monocyte Mac-1 and p150,95 adhesion proteins from an intracellular vesicular compartment to the cell surface. *J Clin Invest* 1987; **80:** 535–544.
18. Dahinden CA, Zingg FE, Maly FE, De Weck AL. Leukotriene production in human neutrophils primed by recombinant human GM-CSF and stimulated with the complement component C5a and FMLP as second signals. *J Exp Med* 1988; **167:** 1281–1295.
19. Rossi AG, O'Flaherty JT. Bioactions of 5-hydroxyeicosatetraenoate and its interaction with platelet activating factor. *Lipids* 1991; **26:** 1184–1188.
20. Serhan CN, Radin A, Smolen JE, Korchak H, Samuelsson B, Weissmann G. Leukotriene $B_4$ is a complete secretagogue in human neutrophils: a kinetic analysis. *Biochem Biophys Res Commun* 1982; **107:** 1006–1012.
21. Baggiolini M, Dewald B. Stimulus amplification by PAF and $LTB_4$ in human neutrophils. *Pharmacol Res Commun* 1986; **18:** Suppl 51–59.
22. Sun FF, McGuire JC. Inhibition of human neutrophil arachidonate 5-lipoxygenase by 6,9-deepoxy-6,9-(phenylimino)-delta$^{6,8}$-prostaglandin $I_1$ (U-60257). *Prostaglandins* 1983; **26:** 211–221.
23. Gillard J, Ford-Hutchinson AW, Chan C, *et al.* L-663,536 (MK-886), a novel, orally active leukotriene biosynthesis inhibitor. *Can J Physiol Pharmacol* 1989; **67:** 456–464.
24. Roberts PJ, Pizzey AR, Khwaja A, Carver JE, Mire-Sluis AR, Linch DC. The effects of IL-8 on neutrophil fMetLeuPhe receptors, CD11b expression and metabolic activity, in combination with other cytokines. *B J Haem* (in press).
25. Powell WS. Rapid extraction of arachidonic acid metabolites from biological samples using octadecylsilyl silica. *Methods Enzymol* 1987; **86:** 467–483.
26. Roberts PJ, Devalia V, Faint R, *et al.* Differentiation-linked activation of the respiratory burst in a monocyte cell line (U937) via FcγRII. *J Immunol* 1991; **147:** 3104–3115.
27. Bauldry SA, McCall CE, Cousart SL, Bass DA. Tumor necrosis factor-α priming of phospholipase $A_2$ activation in human neutrophils. *J Immunol* 1991; **146:** 1277–1285.
28. Ford-Hutchinson AW. FLAP: a novel drug target for inhibiting the synthesis of leukotrienes. *Trends Pharmacol Sci* 1991; **12:** 68–70.

ACKNOWLEDGEMENTS. This work was financially supported by the Kay Kendall Leukaemia Fund.

**Received 9 February 1993; accepted in revised form 11 March 1993**
